# A computational study of the impact of inhomogeneous internodal lengths on conduction velocity in myelinated neurons

**DOI:** 10.1371/journal.pone.0191106

**Published:** 2018-01-12

**Authors:** Abby Scurfield, David C. Latimer

**Affiliations:** Department of Physics, University of Puget Sound, Tacoma, Washington, United States of America; Yale University School of Medicine, UNITED STATES

## Abstract

Age-related decreases in the conduction velocity (CV) of action potentials along myelinated axons have been linked to morphological changes in the myelin sheath. In particular, evidence suggests the presence of segmental demyelination and remyelination of axons. In remyelinated segments, the distance between adjacent nodes of Ranvier is typically shorter, and myelin sheaths are thinner. Both experimental and computational evidence indicates that shortened internodes slows CV. In this computational study, we determine the impact of progressive segmental demyelination and remyelination, modeled by shorter internodes with thinner myelin sheaths interspersed with normal ones, upon the CV. We find that CV progressively decreases as the number of remyelinated segments increases, but this decrease is greater than one would expect from an estimate of the CV based merely upon the number of short and long internodes. We trace the additional suppression of the CV to transitions between long and short internodes. Our study presents an important consideration for the precise modeling of neural circuits with remyelinated neurons.

## Introduction

Action potentials (AP) are electrical signals propagated distally along the axon, prompted by an influx of current into the cell, and at synapses they result in the timely release of neurotransmitters necessary for neural communication. The timing of these signals depends, in part, on the conduction velocity (CV) of the AP along the axon. For an unmyelinated neuron, conduction velocity is determined by the electrical properties of the intracellular medium and the selectively permeable membrane which contains voltage-gated ion channels. When an influx of charge in an otherwise quiescent axon results in a transthreshold depolarization, an AP is generated. This phenomenon can be effectively modeled via a linear core-conductor model with membrane dynamics described by the Hodgkin-Huxley equations [1]. For unmyelinated neurons, conduction velocities roughly scale as $\sqrt{d\;{\left[\mu \text{m}\right]}^{-1}}$ m/s where *d* is the diameter of the axon, so for, say, a 500 *μ*m diameter squid giant axon, the CV is roughly 22 m/s, consistent with experimental measurements [1, 2].

The conduction velocity of a myelinated axon can be a factor of ten (or more) greater than that of a geometrically similar unmyelinated axon. Myelinated neurons make up a large proportion of all neurons in the human body, more so in the central nervous system (CNS). Specifically, all peripheral nervous system (PNS) neurons with diameters greater than around 1 *μ*m and all CNS neurons with diameters greater than around 0.2 *μ*m are myelinated [3–5]. In a myelinated neuron, the axon’s active membrane is confined to a periodic array of small nodes of Ranvier in between longer internodal segments that are wrapped in an insulating myelin sheath. While the mechanisms of PNS and CNS myelination are different, both Schwann cells and oligodendrocytes wrap their cytoplasm in consecutive layers around axons, leading to important electrical changes that result in the increased signal speeds recorded from myelinated neurons [6]. The layering of lamellae results in an increased effective resistance and decreased effective capacitance of the axonal membrane at the internodes, decreasing transmembrane currents in these areas, allowing an electrical signal to impact more distal regions of the axon. As a result, APs propagate rapidly along the axon in a saltatory manner, jumping from node to node; CV is increased relative to an unmyelinated fiber because there is less active membrane to excite. Data indicate that the CV is roughly proportional to fiber diameter, scaling as 6*D*[*μ*m]^−1^ m/s for some axons, but this scale factor does vary [7–10]. The complex factors impacting the CV in myelinated axons can be found in Refs [11–13], including, for instance, myelin thickness and capacitance.

An age-related decrease in the CV along myelinated axons has been observed [14–16], and this decrease has been linked to structural changes in the myelin sheath [17, 18]. With age, some lamellae split to form pockets of dense cytoplasm, leading to both structural and functional degeneration. Additionally, continued myelin production can lead to thicker myelin sheaths or redundant myelin. In old rhesus monkeys, there is also evidence of myelin degeneration and then remyelination in the CNS, as indicated by the increase in the number of oligodendrocytes and axonal nodes and paranodes [19]. When axons undergo demyelination, the axon remains intact while segments of myelin are progressively destroyed. In many cases, due to bio-signaling attempts to correct the deficit, axons undergo remyelination to reinstate some degree of the insulation that was lost [20].

Remyelination is triggered by complex signaling pathways, causing the proliferation of both oligodendrocyte progenitors and Schwann cells which then attach themselves to demyelinated membrane [21, 22]. Many studies have shown that remyelination is not a substitute for the original myelin, as conduction velocities of remyelinated neurons are much slower than they were before insult, both in the CNS and PNS [23–26]. In fact, the slowing of conduction velocity in feline spinal cord axons after injury and remyelination is a direct result of shorter internodes [24]. Multiple additional studies have supported this finding of shorter internodes upon remyelination [27], along with the observation of thinner internodes [28–32]. Injured mouse neurons have, upon remyelination, internode lengths around 55% of the length of a healthy internode [24]. Human studies show similar age-related changes in internode length. Relative to younger humans, older humans have a greater range of internode lengths for axons of a given diameter with a significant number of shorter internodes present. Shorter internodes were roughly 50% the size of those expected in younger subjects, but some were as small as 30% of the typical internode length in younger subjects [33, 34]. Furthermore, in Ref [33], through the analysis of single fibers, the authors find evidence of Wallerian degeneration in which internodes are all uniformly smaller and, additionally, segmental demyelination in which segments of shorter internodes exist adjacent to those of normal length.

Neural communication is dependent upon the coordination of circuitry, and therefore can be interrupted by minor changes in signal speeds [35]. The timely arrival of depolarizing signals at the postsynaptic neuron is an important factor in creating proper signal summation to initiate the next action potential [36]. When signals are slowed by just 10%, they can fail to integrate and communication may be terminated [36]. In older rhesus monkeys, the prevalence of shorter internodes has been correlated with cognitive decline perhaps due to the decreased CV [19]. If age-related remyelination with shorter, thinner internodes is progressive as suggested by the data in Refs [33, 34], then we expect the decrease in conduction velocity to also track with age, possibly impacting cognition in a progressive manner.

In this study, we use a computational model to investigate the degree to which conduction velocity is impacted by progressive segmental demyelination and remyelination in an axon. Specifically, we create a class of model axons in which a fraction of the healthy internodes have been replaced by shorter internodes of approximately one-half the original length, simulating a remyelinated segment. The remyelinated segments are randomly positioned, subject to a binomial distribution with remyelination fraction *p*. We find that the conduction velocity decreases as *p* increases as expected, but the CV is generally lower than one might naively expect. The conduction velocity not only depends on the absolute number of short and long internodes but also their distribution. For a given *p*, CV is lower for axons with more transitions between long and short internodes, so a random distribution of remyelinated segments would result in a lower CV than a semi-uniform fiber with the same number of remyelinated segments.

## Methods

### The McIntyre, *et al*., model

We employ the computational model of McIntyre, *et al*., for mammalian, myelinated motor neurons [37]. The model has a double-cable structure with an explicit representation of the nodes of Ranvier, paranodes, juxtaparanodes, and internodes. Only the nodes have an active membrane with fast sodium, persistent sodium, slow potassium, and linear leakage channels. In the internodes, there is an explicit representation of the myelin sheath surrounding a passive internodal axolemma and small periaxonal space, yielding a double cable. To accurately represent fiber morphology, the model also includes a myelin attachment segment. Inherent to any computational study are limitations in the abilities of the chosen model to accurately represent a biological equivalent. All model parameters, however, are based in previous experimental work and accurately reproduce prior excitation patterns, including both depolarizing and hyperpolarizing afterpotentials, observed in these studies. All computations were made in the NEURON simulation environment because it provides a customizable and user-friendly interface for computationally efficient modeling of complex neural systems (https://senselab.med.yale.edu/modeldb/showModel.cshtml?model=3810) [38].

In Ref [37], the authors generate nine model axons with fiber diameters ranging between 5.7 *μ*m and 16 *μ*m. For our computations, we choose a model axon in the middle of this range, setting the fiber diameter to 10 *μ*m. We use the default morphological and electrical model parameters for this diameter, except when we change internode length for remyelinated segments. In particular, the distance between adjacent nodes for the (default) normal internode segment is 1150 *μ*m. For a remyelinated segment, we replace one such normal internode with two segments whose internode length is adjusted so that the nodal separation is halved to 575 *μ*m, leaving all other parameters fixed. In effect, the remyelinated internodal segment is 45% the length of the normal internodal segment.

Our base (normal) axon consists of 121 nodes and 120 internodes. For this axon, we initiate an action potential with a model stimulating electrode located at node 11 so as to avoid any boundary effect. Model recording electrodes are placed at nodes 31 and 101. They record the time at which the transmembrane potential first reaches −40 mV, allowing us to compute the conduction velocity between these two points. For model axons with remyelinated segments, we leave fixed the physical position of the stimulating and recording electrodes. For example, in the case in which the entire axon is remyelinated, there are a total of 241 nodes and 240 internodes; the stimulating and two recording electrodes are at nodes 21, 61, and 201, respectively. For all simulations, we use a stimulus amplitude equal to twice the threshold value for a fully remyelinated model; this threshold was determined to be 1.8 nA for a duration of 0.1 ms. A study of the impact of spatial discretization for a double-cable model neuron was performed in Ref [39], and it was found that model results are particularly dependent upon the spatial step size in segments adjacent to the node. In the NEURON environment, spatial discretization is controlled by a parameter called nseg. For all model segments, we found that setting nseg = 27 was sufficient for full convergence. For reference, with nseg = 27 throughout, the largest spatial step size in model is in the the internode at 6.5 *μ*m. All other parts of the axon have a smaller spatial step size. In particular, the node’s step size is 0.04 *μ*m; the paranode’s step size is 0.11 *μ*m; and the juxtaparanode’s step size is 1.7 *μ*m. Also, we used fixed time step integration with a time step of 5 × 10^−4^ ms. Halving this time step changed the conduction velocity by 0.4% but significantly increased computation time. In our simulations, we compute the conduction velocity for a model neuron in which a fraction *p* of the healthy internodes have been replaced by two shorter internodes. For a given *p*, we construct 500 model axons; in each, the segments which are remyelinated are randomly chosen according to the binomial distribution.

### The Gow and Devaux tight junction model

In a study of myelinated axons in the human brain, most were found to have a diameter less than 1 *μ*m [40]. Because we are interested in CNS myelinated fibers, we must also examine axon fibers with diameters smaller than those modeled by McIntyre, *et al*. Traditional double-cable models, as in Ref [41], do no accurately describe the conduction velocity of APs along nerve fibers with diameters less than 0.9 *μ*m without the inclusion of tight junctions (TJs) [42]. Modeling TJs as a series resistance with the myelin membrane better reproduces experimental data for small caliber myelinated neurons in murine optic nerves [42, 43]. The electrical impact of TJs is to lessen capacitive charging of the myelin sheath, and this increases CV in small caliber fibers relative to a traditional double-cable model.

Using Gow and Devaux’s TJ model [43], we compute the impact of partial remyelination upon conduction velocity for a smaller caliber CNS axon. To facilitate comparisons between the TJ and McIntyre, *et al*., models, we choose a fiber diameter that results in an axon which is geometrically similar to the larger caliber fiber. For a 1 *μ*m diameter axon, the distance between adjacent nodes in the TJ model axon is 163 *μ*m. The fiber that we study with the McIntyre, *et al*., model has a similar ratio of node-to-node distance to axon diameter, namely, 167. Also, for the McIntyre, *et al*., model neuron, the ratio of axon to fiber diameter, the so called *g*-ratio, is 0.69. Again, for ease of comparison, we use a *g*-ratio of 0.69 in the TJ model. This *g*-ratio is consistent with measured values for 1 *μ*m diameter axons in the mouse cerebullum, 0.7 [32], and cat optic nerves, 0.77 [31].

The Gow and Devaux TJ model has explicit representations of nodes, paranodes, juxtaparanodes, and internodes. One stark difference between the McIntyre, *et al*., model and that of Gow and Devaux is that in the TJ model the relative size of the juxtaparanode is much larger. For a 1 *μ*m diameter axon in the TJ model, one juxtaparanode has a length equal to about 13% of the node-to-node length, while the internodal segment is about 67% of the node-to-node length. Given this, in the remyelinated internodal segments, we reduce the size of both juxtaparanodes and the internode by 47% so that the node-to-node distance is reduced by 50%. Our computational simulation to assess the impact of partial remyelination upon conduction velocity is identical to the one used with the McIntyre, *et al*., model, and it is also executed in the NEURON simulation environment. As before, we use a fixed time step of 5 × 10^−4^ ms. Spatial discretization for each segment was modified until solutions converged. This occurred when nseg was 13 for the nodes (step size of 0.1 *μ*m), 5 for the paranodes (step size of 0.2 *μ*m), 5 for the juxtaparanodes (step size of 4 *μ*m), 9 for the internodes (step size of 12 *μ*m). We used a stimulating current of 0.72 nA, twice the threshold current for a completely remyelinated fiber.

With the TJ model we also examine separately the impact that remyelinated segments with thinner myelin sheaths have on conduction velocity. The measured *g*-ratio for 1 *μ*m diameter remyelinated axons in the mouse cerebullum is 0.88 [32] and in cat optic nerves is 0.84 [31]. We average these and assume the *g*-ratio for the thinner remyelinated internodes to be 0.86.

## Results and discussion

### Simulation results and naive conduction velocity estimates

**Fig 1:**
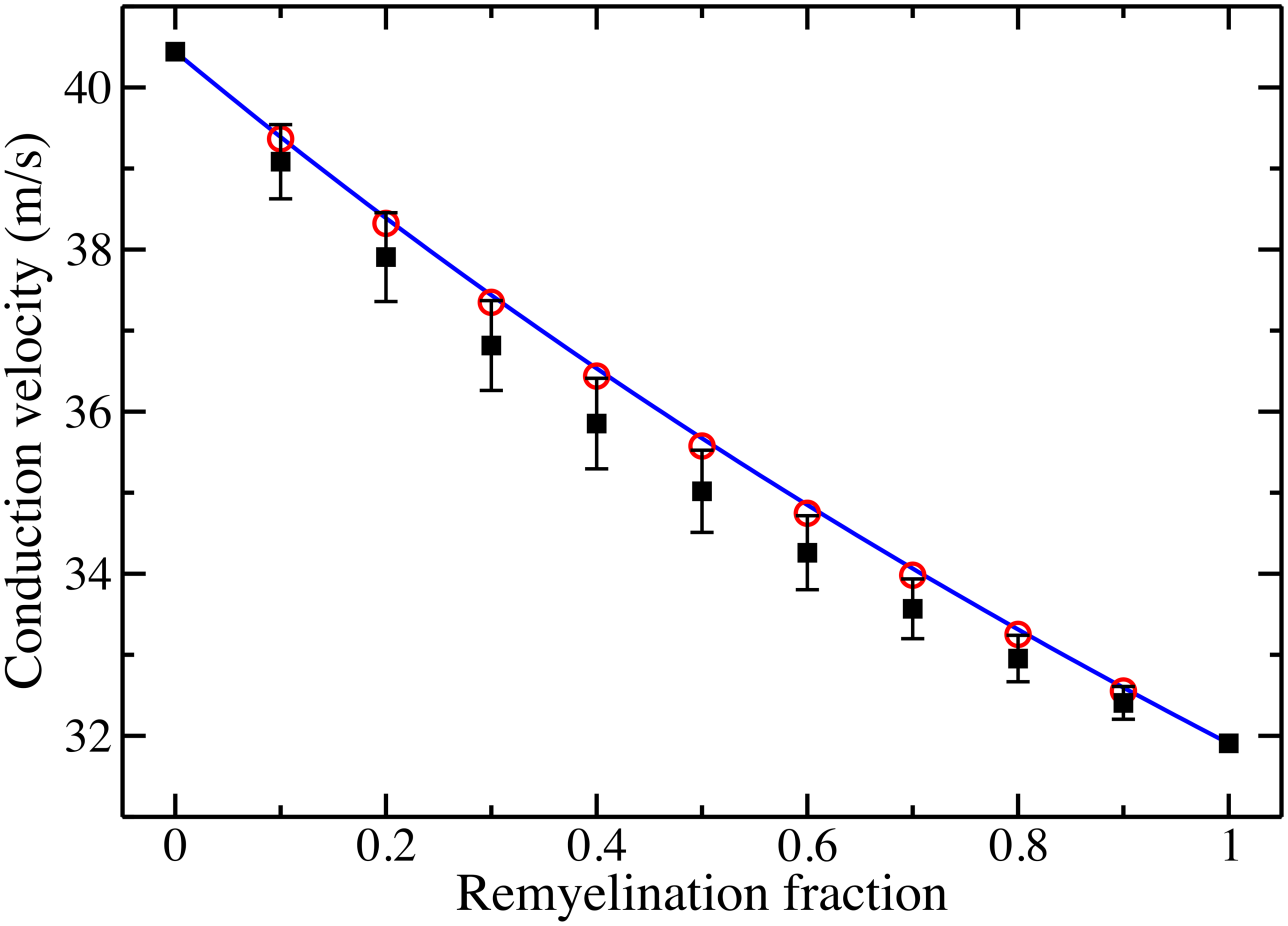
Conduction velocity along progressively remyelinated axons. The black squares represent the average CV (and standard deviation) for 500 model neurons with a given fraction of remyelinated segments. The blue solid curve is the benchmark velocity $\bar{v}\left(p\right)$. The red circles represent the CV for an axon with uniform internode length given by the average internode length of the remyelinated model neurons.

We present the results of our simulations for the McIntyre, *et al*., model in Fig 1. For a given fraction of remyelination *p*, the average conduction velocity is depicted by the black squares. For *p* = 0, the axon consists fully of segments with a (normal) nodal separation of *ℓ* = 1150 *μ*m; the conduction velocity is determined to be *v*_*ℓ*_ = 40.44 m/s. For *p* = 1, the axon fully consists of remyelinated segments with a nodal separation of $\frac{\ell }{2}=575$ *μ*m; the conduction velocity is determined to be $v_{\frac{\ell }{2}}=31.91$ m/s.

En route to understanding the results of our simulation, we establish a simple benchmark velocity, $\bar{v}\left(p\right)$, for comparison. Based upon the velocities in the uniform fibers above, we can compute the travel time across a single internode of length *ℓ* and $\frac{\ell }{2}$. The transit time for a single internode in a uniform fiber would be $\ell v_{\ell }^{-1}$ for the normal fiber and $\frac{1}{2}\ell v_{\frac{\ell }{2}}^{-1}$ for the fully remyelinated one. Given this we can formulate a reasonable guess for the CV in a partially remyelinated fiber. Suppose we begin with an axon with *N* normal internodes and then remyelinate a fraction *p* of these. One might assume that the travel time across an individual internode in the inhomogeneous fiber is the same as it would be in the corresponding uniform fiber above. If this were the case, then the naive travel time for an AP across a partially remyelinated axon is $t\left(p\right)=N\left(1-p\right)\ell v_{\ell }^{-1}+Np\ell v_{\frac{\ell }{2}}^{-1}$, yielding a benchmark velocity

$$\begin{array}{c} \bar{v}\left(p\right)≔\frac{N\ell }{t(p)}=\frac{v_{\ell }v_{\frac{\ell }{2}}}{\left(1-p\right)v_{\frac{\ell }{2}}+pv_{\ell }}. \end{array}$$ (1)

This velocity is plotted as the blue solid curve in Fig 1. Relative to the model axons, this velocity is anywhere between 0.7 to 1.3 standard deviations higher than the average model neuron CV.

For fixed axon diameter, the conduction velocity depends nonlinearly on the internode length [44], so it is possible that the deficit between the model axon and benchmark velocities is a consequence of this nonlinearity. To explore this, we also compute, via simulation, the conduction velocity for a *uniformly* myelinated axon whose internode length is given by the average internode length of the remyelinated axon. The results for this computation are shown as the red circles in Fig 1. The CV along these uniformly myelinated fibers closely tracks $\bar{v}\left(p\right)$ and, thus, is consistently high relative to the randomly remyelinated model neurons with inhomogeneous internodal length. As such, the nonlinear dependence of the CV on the internode length does not explain the model data. The failure of these two CV estimates suggests that the *distribution* of inhomogeneous internodal lengths impacts conduction speeds; we confirm this below.

### Velocity estimate in uniformly myelinated axon

To understand the impact of inhomogeneous internodal lengths, we will first recount in this section the conduction velocity estimate for myelinated neurons found in Ref [2] and then modify the arguments as needed to account for a fiber with two different internodal lengths. Assuming that each node of Ranvier is an isopotential, the membrane potential at adjacent nodes can be described via a discrete cable equation. Assuming a traveling wave solution to this equation, Keener and Sneyd are able to relate, via approximation, the discrete cable equation to the cable equation describing a traveling wave along an unmyelinated fiber. We summarize their argument below.

Our discussion begins with the cable equation for the membrane potential *V*(*x*, *t*) in an unmyelinated axon

$$\begin{array}{c} C_{m}\frac{\partial V}{\partial t}+I_{\text{ion}}=\frac{d}{4R_{c}}\frac{\partial^{2}V}{\partial x^{2}}. \end{array}$$ (2)

Here, *C*_*m*_ is the membrane capacitance per unit area; *d* is the axon diameter; *I*_ion_ is the membrane ionic current per unit area; and *R*_*c*_ is the cytoplasmic resistivity in the axon. We assume the extracellular space to be an isopotential. A traveling wave solution with speed *v* has the form *V*(*x*, *t*) = *U*(*ξ*) where *ξ* ≔ *x* + *vt*. Given this ansatz, the cable equation, Eq (2), takes the form

$$\begin{array}{c} \frac{d}{4R_{c}}\frac{d^{2}U}{d\xi^{2}}-C_{m}v\frac{dU}{d\xi }-I_{\text{ion}}=0. \end{array}$$ (3)

Moving to a myelinated axon, the cable equation is appropriate for the nodes of Ranvier, but given their diminutive size, we will approximate the nodes as isopotentials. Because the internodes are wrapped with roughly 100 extra layers of membrane, the membrane capacitance decreases by about a factor of 100 while its resistance increases by the same factor. Given this, and the few ion channels in the internodal region of the axon, the membrane potential satisfies ∂^2^ *V*/∂*x*^2^ = 0. That is, between nodes *n* and *n* + 1, the potential is linear $V\left(x\right)=V_{n}+\frac{\left(V_{n+1}-V_{n}\right)}{\ell }x$ with internode length *ℓ*. With this, we can determine the current entering node *n* from an adjacent internode

$$\begin{array}{c} i_{n}=-\frac{\pi d^{2}}{4R_{c}}\frac{\partial V}{\partial x}=\frac{\pi d^{2}}{4R_{c}\ell }\left(V_{n-1}-V_{n}\right). \end{array}$$ (4)

Including these currents from the adjacent internodes, the cable equation for node *n* becomes a discrete cable equation linking the potentials at adjacent nodes

$$\begin{array}{c} \mu \pi d(C_{m}\frac{\partial V_{n}}{\partial t}+I_{\text{ion}})=\frac{\pi d^{2}}{4R_{c}\ell }\left(V_{n+1}-2V_{n}+V_{n-1}\right). \end{array}$$ (5)

where *μ* is the node length.

For an AP traveling along a uniformly myelinated axon, the membrane potential at adjacent nodes will be related via a time delay *τ*, viz., *V*_*n*+1_(*t*) = *V*_*n*_(*t* − *τ*). With the traveling wave ansatz, the discrete cable equation, Eq (5), becomes a delay differential equation. By expanding the delay terms *V*(*t* ± *τ*) in a Taylor series, Keener and Sneyd convert this into an ordinary differential equation [2]

$$\begin{array}{c} C_{m}\frac{\partial V}{\partial t}+I_{\text{ion}}=\frac{d\;\tau^{2}}{4R_{c}\ell \mu }\frac{d^{2}V}{dt^{2}}. \end{array}$$ (6)

Through a change of variables, $\xi :=\frac{\sqrt{\ell \mu }}{\tau }t$, this equation takes on the same structure as the traveling wave equation for an unmyelinated axon, Eq (3),

$$\begin{array}{c} \frac{d}{4R_{c}}\frac{d^{2}V}{d\xi^{2}}-C_{m}\frac{\sqrt{\ell \mu }}{\tau }\frac{dV}{d\xi }-I_{\text{ion}}=0, \end{array}$$ (7)

where $v=\frac{\sqrt{\ell \mu }}{\tau }$ is the membrane-dependent speed that yields a (finite) physical solution to the differential equation. Assuming the size of the node is negligible, *μ* ≪ *ℓ*, one can approximate the conduction speed in the uniformly myelinated axon (with internode length *ℓ*) to be

$$\begin{array}{c} v_{\ell }=\frac{\ell }{\tau }=\sqrt{\frac{\ell }{\mu }}v. \end{array}$$ (8)

From this crude estimate, we see that the conduction speed in a myelinated neuron scales with the square root of the internode length.

In particular, this calculation predicts that the wave speed in an axon with internode length *ℓ* should be greater than a similar axon with internode length $\frac{\ell }{2}$ by a factor of $\sqrt{2}\approx 1.41$. In our simulations, we find a speed ratio of $v_{\ell }/v_{\frac{\ell }{2}}=1.27$. The ratio estimate has the right order of magnitude but differs from our explicit computation by about 10%.

### Velocity estimate in a semi-uniformly myelinated axon

We now adapt the arguments of Keener and Sneyd to a myelinated axon with non-uniform internodal length. For simplicity, we begin with a semi-uniform fiber, half of which has uniform internodes of length *ℓ*_*a*_ and the other half of which has uniform internodes of length *ℓ*_*b*_. The transition between these two uniform regions occurs at node *n*. We will focus on developing an equation that describes the membrane potential at this node, which is adjacent to internodes of length *ℓ*_*a*_ and *ℓ*_*b*_. To be concrete, we will assume that intracellular current entering node *n* comes from a myelinated segment with internode length *ℓ*_*a*_, and the intracellular current leaving the node enters a segment of internode length *ℓ*_*b*_. The discrete cable equation for this node is

$$\begin{array}{c} C_{m}\frac{\partial V_{n}}{\partial t}+I_{\text{ion}}=\frac{d}{4\mu R_{c}}(\frac{V_{n+1}-V_{n}}{\ell_{b}}+\frac{V_{n-1}-V_{n}}{\ell_{a}}). \end{array}$$ (9)

For node *n*, we will assume *V*_*n*_(*t*) = *V*_*n* − 1_(*t* − *τ*_*a*_) = *V*_*n*+1_(*t* + *τ*_*b*_) for times *τ*_*a*,*b*_ to be determined. We now Taylor expand the potential in adjacent nodes up to second order in *τ*_*a*,*b*_; the discrete cable equation for node *n* becomes

$$\begin{array}{c} C_{m}[1+\frac{d}{4\mu R_{c}C_{m}}(\frac{\tau_{a}}{\ell_{a}}-\frac{\tau_{b}}{\ell_{b}})]\frac{\partial V_{n}}{\partial t}+I_{\text{ion}}=\frac{d}{8\mu R_{c}}(\frac{\tau_{a}^{2}}{\ell_{a}}+\frac{\tau_{b}^{2}}{\ell_{b}})\frac{\partial^{2}V_{n}}{\partial t^{2}}. \end{array}$$ (10)

This equation is not on the same footing as the approximated delay differential equation, Eq (6), for the uniformly myelinated fiber because there is no fixed delay relating the potential at adjacent nodes. Evidence of this fact is the appearance of the two unknown travel times *τ*_*a*,*b*_. But, perhaps the equation can still give us a qualitative hint as to how inhomogeneities can impact the propagation speed. With a change of variables $\xi =\sqrt{2\mu }{\left(\frac{\tau_{a}^{2}}{l_{a}}+\frac{\tau_{b}^{2}}{l_{b}}\right)}^{-\frac{1}{2}}t$, Eq (10) can formally resemble the traveling wave equation for an unmyelinated fiber, Eq (3), with speed

$$\begin{array}{c} v=\sqrt{2\mu }{\left(\frac{\tau_{a}^{2}}{l_{a}}+\frac{\tau_{b}^{2}}{l_{b}}\right)}^{-\frac{1}{2}}\left[1+\frac{d}{4\mu R_{c}C_{m}}\left(\frac{\tau_{a}}{l_{a}}-\frac{\tau_{b}}{l_{b}}\right)\right]. \end{array}$$ (11)

Ultimately, we would like to determine the propagation velocity across the axon segment surrounding node *n*, *v*_*a*+*b*_ = (*ℓ*_*a*_ + *ℓ*_*b*_)/(*τ*_*a*_ + *τ*_*b*_), but this is impossible because we have two unknown quantities *τ*_*a*,*b*_.

In order to make some inroads on this problem, we will make two assumptions. First, we assume that the term in square brackets in Eq (11) does not deviate too much from unity; second, we will assume that *τ*_*b*_ is what one would naively expect in a uniformly myelinated axon, namely, $\tau_{b}=\frac{\sqrt{\ell_{b}\mu }}{v}$. We can then perturbatively estimate the conduction speed across this segment of myelin by solving for *τ*_*a*_. We anticipate that this time does not differ greatly from a naive estimate (which can be justified post hoc), $\tau_{a}=\frac{\sqrt{\ell_{a}\mu }}{v}+\delta \tau_{a}$. Inserting these values into Eq (11), we find, to leading order, the percent change in propagation time across the internode of length *ℓ*_*a*_ is

$$\begin{array}{c} \frac{\delta \tau_{a}}{\tau_{a}}\approx \frac{v_{a}-v_{b}}{v_{b}}{\left(1-\frac{2\mu R_{c}C_{m}}{d}v_{a}\right)}^{-1}, \end{array}$$ (12)

where $v_{a,b}\approx \sqrt{\frac{\ell_{a,b}}{\mu }}v$. To be explicit, it is the quantity *δτ*_*a*_ that dictates whether the conduction velocity deviates from its benchmark value.

Before examining how well this fits with our model calculations, we make one qualitative observation about Eq (12). First, if *v*_*b*_ > *v*_*a*_, then *δτ*_*a*_ is negative; that is, the time for the AP to transit the internode before the *n*th node is less than it would be in a uniformly myelinated neuron. In particular, if an AP propagating along a myelinated axon with internodal lengths $\frac{\ell }{2}$ encounters a region of the axon consisting of longer internodes, say length *ℓ*, then we expect the propagation speed to increase relative to the benchmark speed. The converse is also true.

Through explicit computation, we qualitatively confirm these features; furthermore, we find good quantitative agreement with the change in conduction velocity implied by Eq (12). Recall the CV in a uniform (normal) axon was found to be *v*_*ℓ*_ = 40.44 m/s while the speed in a uniformly remyelinated axon was $v_{\frac{\ell }{2}}=31.91$ m/s. As above, we consider a semi-uniform myelinated axon, one half of which consists of uniform internodes of length *ℓ* and the other half of which consists of uniform internodes of length $\frac{\ell }{2}$. In the situation in which the AP propagates from the longer internodes to the short, our simulation yields a propagation velocity of $v_{\ell \to \frac{\ell }{2}}=35.51$ m/s. For the reversed orientation with the AP going from shorter to longer internodes, we find $v_{\frac{\ell }{2}\to \ell }=35.78$ m/s. Our benchmark estimate of the propagation velocity is $\bar{v}\left(p=0.5\right)=35.67$ m/s. For a more refined estimate, we evaluate Eq (12) for the 10 *μ*m-diameter fiber in our simulations. From Ref [37], for this fiber diameter, the nodes of Ranvier have diameter *d* = 3.3 *μ*m and length *μ* = 1*μ*m; the cytoplasmic resistivity is *R*_*c*_ = 70*Ω*cm and membrane capacitance (per unit area) is *C*_*m*_ = 2 *μ*F/cm^2^. For the situation in which the AP travels from the longer to shorter internodes, the fractional change in propagation time across an internode at the middle of the axon is $\frac{\delta \tau_{a}}{\tau_{a}}\approx 0.41$ resulting in an estimated propagation speed of $v_{\ell \to \frac{\ell }{2}}=35.49$ m/s. For the reverse situation, our formula estimates $\frac{\delta \tau_{a}}{\tau_{a}}\approx -0.29$ so that $v_{\frac{\ell }{2}\to \ell }=35.75$ m/s. Eq (12) predicts the deviations from the benchmark speeds with reasonable accuracy. Though the deviations are small, they can compound in axons with a greater number of transitions between long and short internodes, as the ones considered in our computational study.

### Conferring with results

The preceding discussion about the transition between two uniform regions with differing internodal lengths informs our calculations of the model neuron conduction velocities. Referring to Fig 1, for a given fraction of remyelinated segments, the spread in the conduction speeds for the model neurons is due to two effects. The first is merely statistical, a consequence of the inherent spread of the binomial distribution. The second is due to relative ordering of short and long internodes in a model axon; this ordering determines the number of transitions between segments with different internode lengths.

For the statistical aspect, given a fraction of remyelinated segments *p* and total number of initial (normal) internodes *N*, the binomial distribution on average yields *Np* segments which are demyelinated then remyelinated. But, there is a spread in the distribution; its standard deviation is $\sigma_{\text{bi}}=\sqrt{Np(1-p)}$. As a result, a sizable fraction of model neurons can deviate from the mean number of remyelinated segments by ±*σ*_bi_. This will result in lower (higher) propagation speed relative to the expected $\bar{v}\left(p\right)$ because there are greater (fewer) shorter internodes.

For an explicit example, we consider the most extreme case with *p* = 0.5. In our simulations, we use *N* = 70 (normal) internodes to compute the conduction velocity, so the average model neuron has *Np* = 35 segments that are remyelinated. The standard deviation of the binomial distribution is *σ*_bi_ ≈ 4.2, so it is quite likely that a model axon will have between 31 and 39 remyelinated segments. If the model neuron contains fewer remyelinated segments, we expect a faster speed and vice versa. The spread in naive propagation speed due to this statistical effect is about 13% slower or faster than the expected $\bar{v}\left(p=0.5\right)$. This spread in propagation speeds due to the sampling of the binomial distribution closely matches the spread in velocities for the model neurons.

Though the spread in propagation speeds, Fig 1, is largely an issue of statistical sampling, the overall depression of the mean speed relative to the benchmark expectation $\bar{v}\left(p\right)$ is a consequence of the random distribution of inhomogeneous internodal lengths in the axon. In the previous section, we observed that if an action potential travels across an internode of length *ℓ* to one of length $\frac{\ell }{2}$ then the AP is slowed a bit at the node adjoining the otherwise homogeneous regions; conversely, the AP slightly speeds up when traveling from a shorter to longer internode. In an axon which is randomly remyelinated with shorter internodes, then the number of transitions from shorter to longer internodes is, on average, balanced by those from long to short, but the overall impact of the transitions is a slowing of the AP propagation speed due to a slight asymmetry in the transition effect.

To be concrete, we will explore this idea by focusing again upon the *p* = 0.5 model neurons. From the sample of 500 neurons, we consider only those with excatly *Np* = 35 remyelinated segments so as to remove any confounding effects due to the binomial statistics. Of those 50 model neurons, we find that the conduction speed and standard deviation is 35.05 ± 0.16 m/s. In this restricted sample, on average the number of transitions from long to short internodes is roughly 17, and we find 18 short to long transitions. From our simulation of the semi-uniform axons, we estimate that the time for the conduction of an AP along the axon is increased by roughly 10.2 *μ*s at a long-to-short transition node and decreased by an amount of roughly 6.8 *μ*s in a short-to-long transition node. For the average number of transitions observed in our restricted sample of model neurons, we then estimate that the propagation speed through such an axon should be 34.87 m/s. Though our estimate is slightly lower than the average value obtained for our model neurons, it does give the correct order of magnitude deviation from the benchmark speed $\bar{v}\left(p=0.5\right)=35.67$ m/s. Similar results hold for the other remyelinated neurons that we considered.

### Impact of tight junctions and thinner myelin sheaths

**Fig 2:**
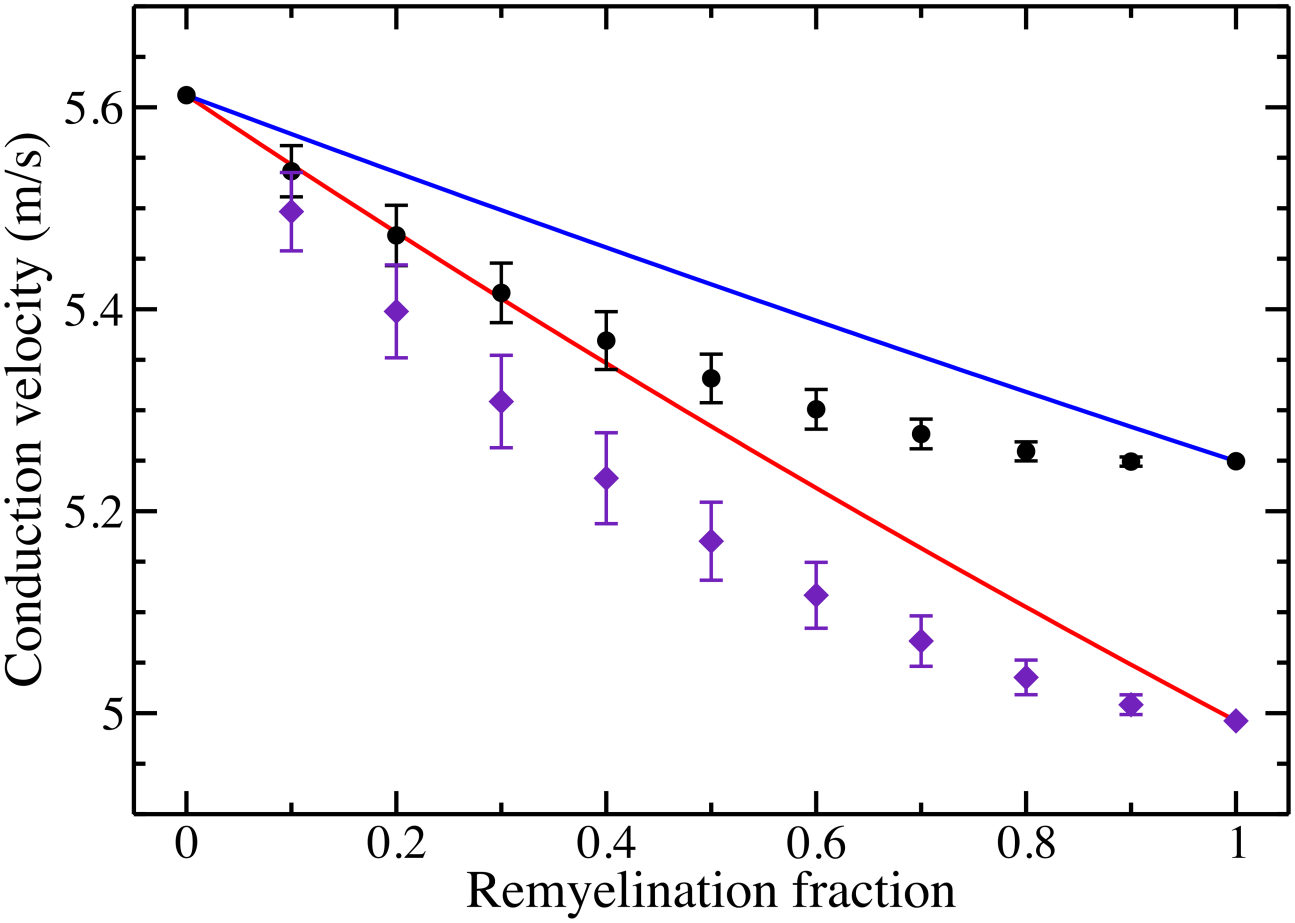
Conduction velocity along progressively remyelinated axons for the Gow and Devaux TJ model. The black circles represent the average CV (and standard deviation) for 500 model neurons with a given fraction of remyelinated segments with a *g*-ratio of 0.69; the blue solid curve is the benchmark velocity $\bar{v}\left(p\right)$ for this case. The purple diamonds represent the average CV (and standard deviation) for 500 model neurons with a given fraction of remyelinated segments whose *g*-ratio is 0.86; the red solid curve is the benchmark velocity $\bar{v}\left(p\right)$ for this case.

To model a 1 *μ*m diameter axon, we turn to the Gow and Devaux TJ model [43]. In Fig 2, we plot, as black circles, the average CV for 500 model neurons with given remyelination fraction *p*, assuming a *g*-ratio of 0.69. For *p* = 0, the axon consists fully of segments with a (normal) nodal separation of *ℓ* = 163 *μ*m; the conduction velocity is determined to be *v*_*ℓ*_ = 5.61 m/s. For *p* = 1, the axon consists fully of remyelinated segments with a nodal separation of $\frac{\ell }{2}=82$ *μ*m; the conduction velocity is determined to be $v_{\frac{\ell }{2}}=5.25$ m/s. For both the normal and remyelinated axons, the decrease in conduction velocity is as expected compared to the 10 *μ*m fiber; that is, the velocity scales linearly with fiber diameter [4, 7]. For the 1 *μ*m diameter axon, the fiber diameter is 1.4 *μ*m. Compared to the 10 *μ*m fiber, this results in the overall observed seven-fold decrease in CV. In Fig 2, we also plot as the solid blue curve the benchmark velocity based purely upon the number of short and long internodes. Qualitatively, the CV for the model neurons is much lower than naive expectations, consistent with the results derived from the McIntyre, *et al*., model.

For the same model neurons, we also explore the impact of tight junctions. To include TJs in their model, Gow and Devaux include an additional resistance, of resistivity 600 *Ω* cm^2^, in series with the myelin membrane [43]. If this resistivity is reduced to 60 *Ω* cm^2^, then the model effectively lacks TJs. We ran our simulations for the Gow and Devaux model without TJs. We found that the CV was much lower than the model with TJs. Furthermore, the conduction velocity was relatively independent of the remyelination fraction. For normal internodal lengths, the CV was 4.29 m/s, and for a fully remyelinated fiber, the CV was about 1% higher, namely 4.35 m/s. This is at odds with the analytical estimate, Eq (8), that indicates that the velocity should scale as the square root of internode length. In that analytical work, we assumed no transmembrane current in the internodal region. It is likely that the smaller axons lacking TJs violate this assumption, but tight junctions, at least in part, reduce current flow through the myelin which better approximates the idealized axon in the analytical work.

Finally, with the Gow and Devaux model, we explore one further change in the myelin sheath that occurs during remyelination: thinner internodes. We run our simulations for the 1 *μ*m diameter axons assuming, again, that the normal internodes have a *g*-ratio of 0.69, but for the remyelinated internodes, we set the *g*-ratio equal to 0.86 [31, 32]. This further suppresses the CV in the fully remyelinated fibers resulting in a CV of 4.99 m/s, an 11% decrease from the normal axon. In Fig 2, we plot the CV for a given remyelination fraction, represented by purple diamonds, along with the benchmark speed, depicted by the solid red line. The CVs for the inhomogeneous remyelinated fibers are qualitatively consistent with our previous results. As with the other cases, we find that the distribution of the inhomogeneities in internodal lengths impacts the conduction velocity. Generally, if a fiber has a large number of transitions between long and short internodal lengths, conduction velocity decreases relative to a semi-uniform fiber.

## Conclusion

Through this computational study, we were able to quantitatively assess the impact of progressive segmental demyelination and remyelination, simulated by shorter internodes with thinner myelin sheaths interspersed with normal internodes, on the conduction velocity of action potentials. Previous computational studies have shown that shortened internodes reduce CV [13, 24], consistent with experimental findings [17, 18, 23, 24]. Our results confirm this, but we additionally find that the CV is sensitive to the distribution of shorter internodes relative to the standard longer internodes. In our simulations, we find CVs that are consistently lower than one might expect from an estimate of the CV based merely on the number of short and long internodes. We find that an AP which travels from a shorter to longer internode speeds up relative to expectations, and one which travels from long to short slows. Because of a slight asymmetry in the two cases, the net impact of such transitions is to slow the CV of an AP in an axon that has undergone random segmental demyelination and remyelination. Adapting the analysis of CV for myelinated neurons in Ref [2], we are able to mathematically trace this phenomena directly to the transitions between internodes of different lengths.

The absolute difference between the average CV from our model simulations and the benchmark speed is not relatively large, on the order of 2% at most. But, we recall that the synchrony of timing in neuronal circuits can be disrupted by changes in CV on the order of 10% [36]. For the simulation of the 10 *μ*m diameter fiber, we find that if one-third of the axon has undergone random remyelination then the CV has decreased by this critical factor. From our benchmark speed, we would determine that roughly 40% of the axon would need to be remyelinated to decrease the CV by this figure. For the 1 *μ*m axon with tight junctions and thinner internodes, the relative difference between the normal and fully remyelinated fibers is much smaller than the 10 *μ*m fiber. This partially remyelinated fiber has a 10% decrease in conduction velocity when the remyelination fraction is around 75%, while the naive benchmark velocity suggests that the CV is not slowed to this degree until the remyelination fraction reaches 90%. Based on these results, attention must be paid to the transitions between internodes of different lengths because this difference could be critical in accurately modeling a neural network of neurons with segmental demyelination and remyelination.

We end with a brief comment on the limitations of our study. In our simulations, we study a large diameter PNS axon and a smaller diameter CNS axon (with tight junctions) that is geometrically similar. It is heartening that both axons show qualitatively similar results in regards to the impact of partial remyelination on CV. Our analytical work suggests that the qualitative behavior observed with the model axons will persist for different geometries as long as the myelin is sufficiently insulating, but the actual quantitative details for different fibers can only be reliably accessed through simulation. One other limitation in our study is that our remyelinated segments did not account for the redistribution of ion channels that occurs with morphological changes. In Ref [45], a study of demyelinated axons shows that new sodium channels are produced to meet the added demands of an increased number of nodes in remyelinated segments. After several weeks, the channel density in newly formed nodes is similar to that in established nodes, a result consistent with other studies [46–48]. While the re-establishment of normal channel densities progresses throughout the remyelination process [47, 48], our study assumes completion of this process. As such, our results are only valid for mature remyelinated axons.
